# Acceptability of non-drug therapies in older people with orthostatic hypotension: a qualitative study

**DOI:** 10.1186/s12877-018-0999-5

**Published:** 2018-12-17

**Authors:** Lisa J. Robinson, Ruth M. Pearce, James Frith

**Affiliations:** 1 The Falls and Syncope Service, Newcastle upon Tyne Hospitals NHS Foundation Trust, Newcastle upon Tyne, NE1 4LP UK; 2 Institute of Cellular Medicine, Newcastle University, Newcastle upon Tyne, NE2 4HH UK; 3 The NIHR Newcastle Biomedical Research Centre, Campus for Ageing and Vitality, Newcastle University, Newcastle upon Tyne, UK

**Keywords:** Orthostatic hypotension, Acceptability of healthcare, Qualitative research, Conservative treatment

## Abstract

**Background:**

Orthostatic hypotension (OH) is highly prevalent in older populations. It is associated with a reduced quality of life and an increased risk of dementia, stroke and death. Non-pharmalogical therapies are the recommended first-line therapy and are preferred to drug treatments by older people. However, uptake and adherence is low and evidence for their use is lacking. Objective: Determine the acceptability of non-pharmalogical interventions for OH in older people.

**Methods:**

This qualitative study, nested within a phase II efficacy study, recruited 25 people aged over 60 years from a Falls and Syncope Clinic. All participants had experienced the following non-pharmalogical therapies within a phase II study: bolus water drinking, compression stockings, abdominal compression, physical counter-manoeuvres. Individual semi-structured qualitative interviews were recorded and transcribed verbatim. Emergent themes were identified through framework analysis of transcripts.

**Results:**

Physical counter-manoeuvres were considered the most acceptable therapy as no equipment is required, they can be performed discreetly and are only required during postural change. Bolus water drinking was mostly considered as an acceptable therapy, although there were significant concerns around urinary frequency. The idea of bolus water drinking was a barrier to its uptake, but once experienced it was easier than anticipated. Participants had mixed views on acceptability of abdominal compression whereas compression stockings were considered unacceptable by the majority of participants. This was due to the practicalities of applying/removing the compression and the stigma attached to their appearance.

**Conclusions:**

Current first-line treatment with compression stockings is largely unacceptable to older people with OH, challenging current guidelines. In order to promote uptake and adherence, first line therapy should focus on bolus-water drinking and physical counter-manoeuvres.

## Background

Orthostatic hypotension (OH) results from a sustained reduction in blood pressure (BP, ≥20 mmHg systolic or ≥ 10 mmHg diastolic) within 3 min of standing [1]. It is highly prevalent in the older population, affecting up to 20% of community-dwelling older people [2]. It is a disabling condition, resulting in reduced quality of life and an increased risk of cognitive impairment and death [3–5]. Much of the evidence to support existing treatment options for OH is of poor quality and largely based on cohorts with autonomic failure, creating uncertainty for its management in older populations [6–9]. Non-drug treatments are recommended as first line therapy and older people have a preference for these therapies over pharmacologic agents due to their existing medication burden [10]. However, non-drug interventions are often more complex to deliver, have lower levels of adherence and the evidence for their use is scanty at best [7, 11].

As the older population is rapidly expanding we can expect a growing demand for evidence in this area. However, if an intervention is deemed unacceptable, its effectiveness becomes irrelevant.

## Methods

### Study design

This qualitative study was nested within a phase 2, non-blinded efficacy study which evaluated the safety and efficacy of non-drug interventions in older people with OH [12]. Semi-structured qualitative interviews were conducted to examine how acceptable each of the non-drug interventions were to participants.

### Population

All participants were aged over 60 years and had a clinical diagnosis of OH according to international criteria [1]. To be eligible, the participant’s OH was judged to be secondary to ageing by their referring clinician, thus excluding reversible causes (such as haemorrhage) and specific neurological causes (such as Parkinson’s disease). Participants were excluded if they had a contra-indication to any of the therapies under evaluation (dysphagia, previous aspiration, fluid restriction or unable to wear compression garments). All participants were recruited via the UK’s Newcastle upon Tyne Hospitals Falls and Syncope Service and the UK Clinical Trials Gateway. This was a purposive sample to ensure that all participants had experienced each non-drug intervention.

### Interventions

All participants experienced each of the following therapies, while wearing continuous non-invasive BP monitoring. Researchers and participants were un-blinded to the BP readings:

1. Bolus water drinking: 480 ml of room-temperature tap water, to consume as much as possible within 5-min.
2. Physical counter-manoeuvres: Upon standing upright, participants were encouraged to tense their lower limb and abdominal muscles [13].
3. Compression stockings: Full leg length, grade 2 compression stockings (23–32 mmHg, mediven plus).
4. Abdominal compression: An elasticated belt (promedics pro-tem belt), tightly applied (at 10 mmHg) to the participant’s abdomen and pelvis.

### Demographic data

For the purposes of providing descriptive data of the cohort’s level of co-morbidity, the Charlson Comorbidity Score is reported alongside the number of medications [14]. Symptom severity was captured using the Orthostatic Hypotension Questionnaire [3]. Supine and standing BP were recorded using continuous non-invasive methods (Taskforce, CNSystems) during the quantitative component of the study. These methods are described in more detail elsewhere [12].

### Setting

Twenty of the qualitative interviews were conducted in the participants’ own home, with the remaining five participants choosing to attend the out-patient hospital clinic. To aid recall, interviews were conducted as soon as possible after the efficacy study [median 8 days (interquartile range 7–14)]. The decision to conduct the interview at a separate appointment, rather than during the efficacy study, was primarily to avoid the time burden, but also in the hope that the participants would talk more openly about their experiences in their own homes.

### Interviews

Semi-structured interviews were conducted, recorded, transcribed verbatim and anonymised by an experienced qualitative researcher (RP). The interview schedule consisted of one open question about each intervention, *‘how did you find [intervention] as a potential treatment?’* with follow up questions to be used as prompts if needed. The average length of the interview was 20 min (range 11 to 43) and a copy of the interview schedule can be found in Appendix 1.

### Sample size

The sample size of 25 was determined by considering both the quantitative and qualitative studies within the programme of research. This sample size was anticipated to be large enough to capture a wide variety of rich data.

### Analysis

The verbatim transcripts were subjected to framework analysis [15]. This method of qualitative data analysis consists of a series of distinct, yet highly interconnected, stages which allows for the possibility of both emergent data themes and the explicit use of a priori issues in the analytical framework [16]. Framework analysis is increasingly being used in health services research, and it complemented the aims of this study as we had predefined areas we wished to investigate while remaining open to the emergence of further topics and themes. To promote transparency, the full data analysis process (familiarisation; identifying a thematic framework; indexing; charting; mapping and interpretation) is described sequentially below. However, it is important to emphasise that analysis was an iterative process and did not occur in a linear fashion.

### Data analysis process

#### Familiarisation

Two researchers (RP and LR) developed an initial sense of the data by reading through a sample of the interview transcripts. *Identifying a thematic framework:* The aim of the nested qualitative study was to explore the acceptability of the different interventions. Therefore, it was decided that it would be most beneficial to consider each intervention separately. The main themes to emerge throughout the data (and each intervention) which appeared to help answer the question we were asking were: positive responses, perceived barriers and the associated suggested solutions. *Indexing*: The transcripts were sifted by RP, quotes were highlighted and comparisons were made both within and between the interview data. During this stage, the data were labelled using the three main thematic headings for subsequent retrieval and exploration. *Charting:* Selected quotes were lifted from their original context and re-arranged under the emerging thematic framework. This process provided a valuable stage in helping to manage the data, making sense of what was going on by getting rid of extra and irrelevant data. Whilst charting, RP made note of any changes to the analytical framework. The evolving thematic framework was discussed with LR and JF at regular data analysis meetings. Illustrative quotes were tagged and managed using Word. *Mapping and interpretation:* We considered this data alongside the efficacy data, in order to obtain a richer understanding of the potential for each of these interventions to be both effaceable and acceptable.

## Results

All 25 participants were interviewed, their demographic data are summarised in Table 1. Analysis of the data led to the emergence of three main themes spanning all four of the interventions. They were: 1) *‘Tolerability’* 2) *‘Perceived barriers’* and 3) *‘Potential solutions’*. These themes are explored below, in relation to each intervention and using quotations from the interviews to gain an insight into how acceptable the four interventions were to the participants.

**Table 1:** Participant characteristics

**Demographic**	
Age	74 (60–92)
Male	15 (60%)
Charlson Comorbidity Score	4 (3–8)
Number of regular medications	4 (0–13)
Fludrocortisone (N)	5
Midodrine (N)	3
Hypertension	5 (20%)
Antihypertensive medication^a^	7 (28%)
• Angiotensin-converting-enzyme inhibitor	6
• Beta-blocker (propranolol)	1
Supine BP	
Systolic	128 (21)
Diastolic	75 (13)
Orthostatic BP drop	
Systolic	41 (22)
Diastolic	19 (13)
Orthostatic symptoms	
Total symptom score	6 (0–51)
Dizziness	2 (0–9)

Normally distributed data are presented as mean (with standard deviation). Non-parametric data is summarised as the median (with the range).

^a^Indications for antihypertensive medications included hypertension, previous stroke, ischaemic heart disease and anxiety

### Bolus water drinking

#### Tolerability

Drinking water was generally well tolerated; 18 participants consumed the full 480 ml (median 480 ml [interquartile range 451 to 480 ml)]. Participants felt positive that they would be able to achieve this in their everyday life without too much difficulty.

*“It was just a glass of water” (03).*

*“It was fine” (06).*

*“No problem at all.*. *.” (07).*

*“dead easy” (13).*

Some people had been given advice to drink more water previously, either specifically for OH or in terms of a healthier lifestyle, and had easily adopted this into their daily routine.

*“I manage two glasses full, sometimes three glasses full*. *.*.” *(11).*

*“I drink water all the time.*. *.” (15).*

### Perceived barriers

Some participants were concerned about the concept of drinking a large amount of water in a short period of time.

*“Normally I would think I can’t drink that amount of water.” (06).*

However, a number of them were surprised when they were able to complete it, without too much difficulty.

*“I was pleasantly surprised how it did go down” (018).*

This illustrated how, for some, it was more the idea of drinking the water bolus that was a greater barrier than actually doing it.

There were a couple of participants who, despite being able to complete the task, admitted that they did not particularly enjoy the experience.

*“Tedious – trying to get a whole lot of water down fairly quickly” (08).*

*“I wouldn’t want to do it too many times during one day*. *.*. *one might get fed up of the whole process”(10).*

Another common concern was how it might impact on urinary frequency, thus providing additional problems to contend with.

*“It might make you want to go to the loo more often (or affect you) going somewhere important, like a long service for instance.” (06).*

One participant was resolute that she would not be able to perform this in the future. In the study she managed to drink only half but felt this was her limit. She was, however, the only participant to feel this way.

*“I felt as if I was going to choke*. *.*. *I was finding it difficult swallowing” (01).*

She was, however, the only one of the participants to feel this way.

### Potential solutions

A common suggestion was to add fruit juice or cordial – anything to improve the flavour.

*“Which would make it more palatable/easier to drink*. *.*. *for those who can’t tolerate plain water.” (03).*

Others focused on temperature and suggested it might be easier having a hot drink instead of water.

*“large cup of tea” (01).*

*“Half boiled and half cold.*. *. I find that easier than drinking it cold.” (07).*

Others suggested that helping people understand the benefits of the intervention may help its uptake. Many respondents said that if they knew what a difference it made to their symptoms they would be more motivated to do it.

*“Knowing that the water will make a difference to your symptoms is important.”(14).*

*“I knew I was drinking the water for a purpose, so I think when you’ve got that approach you’re prepared to drink*. *.*. *.” (03).*

### Compression stockings

#### Tolerability

In terms of wearing the stockings, many people were quite favourable, feeling that once they were on, they were comfortable.

*“Quite comfortable and they were no problem at all*. *.*.” *(03).*

*“It was quite easy – wearing them. In fact it wasn’t a relief to take them off.*. *.” (08).*

*“I found them very comfortable to be honest and I’m going to try and get myself a pair” (013)*.

However, for the majority of participants, the difficulty in applying the stockings was a major concern.

#### Perceived barriers

Many participants felt that it would simply be too difficult to attempt applying or removing them without additional help.

*“An absolute pain*. *.*. *you were both struggling to get them on me, if I’d got to put those on myself I dread to think how long it would take” (014).*

*“I thought they were impossible to use. You just can’t get them on” (016).*

*“Putting them on was torture” (26).*

Another significant barrier to wearing the stockings was their appearance. The stockings used were tan in colour and thus, to some, were not aesthetically pleasing.

*“They look awful – from a looks point of view. Yours were really, really thick and very sort of, like an old grannies weren’t they?” (06).*

Side effects of the compression was also identified as a barrier, with one participant experiencing itch whilst wearing them.

*“couldn’t bare them” (015).*

#### Potential solutions

Suggested solutions focused on practical ways to get the stockings on. A number of people suggested using stockings that were less tight. Some people, who had worn similar stockings in the past, suggested that below knee length stockings might be easier than full leg-length, especially in terms of getting them on. A common solution to overcoming the difficulty of getting the stockings on was simply to enlist the help of another person.

*“I’m not convinced one person could do them adequately which means there has to be a helper or attendant as well*. *.*. *I don’t think I could get them on alone” (010).*

More innovative suggestions included designing stockings with a zip to make it easier to get them on while one individual wondered about having stockings that could be inflated, to provide compression once they were already on.

*“In this day and age of technology, I would have thought they would have had some sort of zip up ones, or something like that” (016).*

A solution to the concern about their appearance was to have a variety of colour options, or, to simply wear trousers over them, rather than a skirt or dress. One lady, who had worn stockings in the past, explained how her previous ones had been more acceptable, *“possibly because they were black” (06).* She went on to say that she usually wears trousers, so actually, if the stockings were black, they would be *“fine” (06).*

### Abdominal compression

#### Tolerability

In keeping with leg compression, participants generally found abdominal compression to be comfortable, even promoting a sense of safety. Those who had worn something similar for back pain previously with success were more amenable to attempting abdominal compression. One lady was particularly keen and preferred it to all the other therapies that she tried. As someone who had particularly severe symptoms, she felt that wearing the compression belt made her feel safe.

*“I felt it was quite comfortable.” (03).*

*“It wouldn’t bother us at all, pet” (013).*

*“No problem at all, far easier than the stockings” (014).*

*“feel safe” (07).*

#### Perceived barriers

A number of participants were hesitant about abdominal compression. In a similar vein to the stockings, concern was expressed about the ease of being able to apply compression by themselves.

*“I’m not sure how easy or difficult that was to put on myself” (018).*

*“I couldn’t see how you fastened it, if you were on your own” (06).*

*“You’d be like Scarlett O’Hara getting dressed every morning. I wouldn’t consider it at all” (02).*

Some participants mentioned more specific problems with wearing the binder; one person suffered with an abdominal hernia and therefore found the compression uncomfortable*.*

*“a bit sore” (015).*

*“swollen tummy…so tight” (023).*

An additional concern was the length of time they would be expected to wear it per day.

*“I think if you wore them all day you would be thoroughly fed up with it by the evening” (010).*

#### Potential solutions

One of the main suggestions to help encourage people to wear the binder was that it should be worn for a limited period only, such as when planning to be upright, or going outdoors.

*“A couple of hours max” (020)*.

*“Okay if it was a short period*. *.*. *I don’t think it would be comfortable for too long” (018).*

Although some were concerned about being able to put the binder on themselves, one person felt that putting it on himself would in fact be helpful, applying compression to his own level of comfort.

*“An easier method of putting on and taking off” (08).*

“[self-administration so as not to feel] *straight-jacketed” (014).*

### Physical counter-manoeuvres

#### Tolerability

Of all the interventions under evaluation, the physical counter-manoeuvres were the most popular and for a large number of participants were already being used successfully.

*“They told us that at the falls clinic.*. *.” (015).*

*“no problem at all” (03).*

Convenience was a major advantage to this therapy. No preparation is required, no equipment is needed and they can be done almost anywhere. For example, participants described doing them whilst washing up, on public transport and whilst standing in church.

*“Sitting behind the wheel of a car, standing, walking.*. *.” (08).*

Of note, was the number of people already regularly performing physical counter-manoeuvres, who also reported a noticeable improvement in their symptoms.

*“I do that every morning when I get out of bed, when I stand up out of bed” (011).*

*“It seems to help the blood pressure, yes. The dizziness doesn’t go away completely, but it seems to control it, you know” (015).*

*“I find it does work to an extent. I don’t fall over as much as what I used to. I used to fall over a lot” (016).*

#### Perceived barriers

Few participants expressed difficulty performing the manoeuvres. Those who did, were concerned about becoming unsteady and falling.

*“I felt, if nobody had been there, I could’ve fallen over” (01).*

One participant who was very unsteady felt that she didn’t get much benefit from the manoeuvres. Her daughter, who was present at the interview, was quite adamant that the manoeuvres would put her mother at risk of falls.

*“It’s not the real world when you do it at the hospital” (022).*

#### Potential solutions

Overall, participants felt that physical counter-manoeuvres were relatively simple and easy to adopt into daily routine, therefore there were fewer barriers and solutions to discuss. However, it was suggested that it would be useful if participants had some supervision/training in how to do the exercises to ensure accurate technique.

*“To make sure that people are doing it properly. There’s quite a long learning curve before you get the hang of it, tensing the right muscles … once you’ve learnt the trick it would be fairly easy to do” (010).*

## Discussion

This qualitative study demonstrates that non-drug therapies are not universally acceptable to older people with OH. Physical counter-manoeuvres (PCMs) were judged by this cohort to be largely acceptable, with a number of benefits over the other therapies, but perhaps its main advantage is that no equipment is required. This also means that it can be performed anywhere, at any time, with the added value of being relatively discreet. In contrast to the other therapies it also has the advantage that it only needs to be performed as required, during postural change, with no preparation. Nevertheless, it was not universally popular, with concerns about poor balance during muscle tensing leading to a loss of confidence to perform this without supervision. However, perhaps education and supervision in a clinical setting to increase confidence could promote uptake and adherence in these individuals. Indeed, previous research demonstrating the effectiveness of PCMs in vasovagal syncope, employed continuous BP monitoring during the manoeuvres, as a means of biofeedback. As the BP responses to un-blinded to participants, this biofeedback may have influenced their views of the therapies.

Bolus water drinking was generally considered as an acceptable therapy. Interestingly, the thought of bolus-drinking was a more significant barrier than actually doing it, with some individuals being surprised how easy they found it. This finding may help develop adherence strategies; asking individuals to perform this in the clinic setting may overcome the initial barrier preventing its uptake. However, urinary frequency was a major concern which could limit long-term adherence. Unfortunately this study did not measure changes in urinary frequency, which could have challenged or affirmed these concerns. Some participants felt that longer term motivation would be reduced if this therapy had to be repeated throughout the day. Previous studies have demonstrated that the vasopressor effects of bolus water drinking last for up to 90 min, it would therefore require repeated administration to maintain its effects [17]. A further disadvantage is that it may take up to 20 min before the peak pressor effects are seen, and therefore requires a degree of preparation [17]. Unfortunately it is not known whether flavouring the water would result in the same response. In theory, a change in portal osmolality is all that is required to evoke the pressor response, so water is the preferred liquid [18].

There were mixed views concerning abdominal compression. Its main advantage was in promoting a sense of security but this was not experienced by all participants, with many feeling that it was too difficult to apply and too restrictive to wear. These feelings were also expressed in relation to the compression stockings, but more frequently and to a stronger degree. Of all of the therapies, compression stockings were the least popular, and were largely felt to be unacceptable. In addition to the great difficulties in applying them, there were concerns around side effects such as itching, but also around their appearance. Perceived appearance and social stigma are important barriers to the uptake and adherence of therapies [19]. If compression stockings are being considered, a discussion around these areas may be useful to promote adherence. However, there is little value in recommending a treatment if it is deemed unacceptable by those who need it.

This study has several limitations. The number of participants may be considered as relatively small. Due to the modest sample size it cannot claim to be representative of the population. However, it is the largest of its kind in this population and provides unique insights for clinicians to consider when advising their patients. A further limitation is that participants may have only experienced the therapies in the context of this research study. Had participants attempted to use all the therapies in their daily life their experiences may have been different. However, some of the therapies had been used by participants, or had been recommended to them by their clinician and this emerge from the interviews. On the other hand, experiencing the therapies in the research setting, under professional supervision had its own advantages, such as the experience with water drinking, as described above.

## Conclusions

Based on evidence provided in this study, compression stockings should not be used as first line therapy as they are largely unacceptable. Bolus water drinking and physical counter-manoeuvres are both acceptable but physical counter-manoeuvres may have the advantage because they do not depend on equipment, toileting or advance preparation. As abdominal compression has mixed tolerability, its use could be reserved for those who do not respond to water drinking and counter-manoeuvres.

## Appendix

### Appendix 1 - Interview Schedule

How did you find [intervention] as a potential treatment?

- How easy or difficult did you find it?
- How easy do you think it would be for you to use it regularly in daily life?
  - ○ How do you think it might affect your day to day activities?
- What are your concerns about using it regularly?
  - ○ Are there any situations where you would avoid using it?
- If this treatment helped improve your symptoms, would you consider it as worthwhile?
  - ○ What might help you stick to it in the long term?
- Can you think of any other ways we could help people to stick to it?

## Abbreviations

BP: Blood pressure
OH: Orthostatic hypotension
PCM: Physical counter-manoeuvre
